# Co-Designed Mobile-Based Cognitive Training for Older Chinese Americans: Protocol for a Pilot Randomized Controlled Trial Assessing Feasibility and Acceptability

**DOI:** 10.2196/69303

**Published:** 2025-07-21

**Authors:** Tingzhong Xue, Aybey Amy Wei, Bei Wu, Camilla Sanders, Eleanor Schildwachter McConnell, Hanzhang Xu

**Affiliations:** 1 Elaine Marieb College of Nursing University of Massachusetts Amherst Amherst, MA United States; 2 School of Nursing Duke University Durham, NC United States; 3 Chinese-American Friendship Association of North Carolina Cary, NC United States; 4 Rory Meyers College of Nursing New York University New York, NY United States; 5 Duke University Durham United States

**Keywords:** mHealth, cognitive training, Alzheimer disease and related dementias, experience-based co-design, Asian Americans

## Abstract

**Background:**

Older Chinese Americans are at high risk of dementia, yet they often do not access culturally relevant services/programs to reduce their risks due to issues such as language barriers and transportation. BrainHQ is a mobile-based, effective cognitive training program that can potentially address these barriers and delay cognitive decline in older Chinese Americans.

**Objective:**

We aim to evaluate the feasibility and acceptability of a mobile-based cognitive training intervention co-designed by older Chinese Americans and their adult children.

**Methods:**

We applied an experience-based co-design approach that leverages existing cognitive training features and older Chinese Americans’ prior knowledge, lived experiences, and social norms around dementia to co-develop a cognitive training intervention. We conducted an experience-based co-design workshop with Older Chinese Americans (n=10), and their adult children (n=4) to optimize the cultural and linguistic relevance of the cognitive training intervention. Participants used a journey map to brainstorm challenges they may experience when participating in the intervention. Then, the participants created prototypes of intervention components to address these challenges. Finally, we incorporated these prototypes into the co-designed intervention protocol. A total of 30 participants will be recruited into the intervention study and will be randomly assigned to the intervention or waitlist control group (2:1 ratio). The intervention group will complete the mobile-based cognitive training for between 10 and 15 minutes daily for 12 weeks. The primary outcomes are feasibility and acceptability. Global cognition, mental health, physical functioning, and quality of life will be assessed at baseline, 8, and 12 weeks.

**Results:**

This pilot trial received institutional review board approval (Pro00109934l) in November 2024. We enrolled the first participant in December 2024 and aim to complete enrollment by May 2025. We expect to complete all data collection by September 2025. We will analyze the data and report study findings by February 2026.

**Conclusions:**

This study leverages partnerships with academic, industry, and community stakeholders and provides the groundwork for a large-scale randomized controlled trial to test the efficacy of a mobile-based cognitive training intervention for older Chinese Americans. The co-design workshop served as a feasible, innovative approach to engage with the participants and improve the study design. These findings will enhance the culturally tailored delivery of cognitive training to older Chinese Americans and provide insights for broader implementation, improving their engagement in dementia research.

**Trial Registration:**

ClinicalTrials.gov NCT05355870; https://clinicaltrials.gov/study/NCT05355870

**International Registered Report Identifier (IRRID):**

PRR1-10.2196/69303

## Introduction

The older Asian population aged 65 years and above is one of the fastest-growing groups of older adults in the United States [1]. Among this population, Chinese are the largest group, accounting for 26% of Asian older adults [2]. Although Chinese Americans are often perceived as a “model minority” with a higher average socioeconomic status than other minority groups, the picture is incomplete. Older Chinese Americans are heterogeneous, with over 70% being first-generation immigrants, nearly 20% living below the poverty line (compared to 9% among the general US older adults), and close to half having limited English proficiency [2,3]. Although the prevalence of developing Alzheimer disease and related dementias (ADRD) among Chinese Americans is largely unknown [4], these socioeconomic disadvantages shared by older Chinese Americans not only increase their risk of developing ADRD but also limit their equitable access to effective, culturally responsive, preventive services that promote cognitive health. Currently, older Chinese Americans are disproportionately underrepresented in intervention studies aimed at preventing cognitive decline [5]. The rapidly increasing number of people with ADRD, along with the increasing population of older Chinese Americans, calls for the development of novel, effective strategies tailored to this vulnerable population to promote their cognitive health.

Cognitive training is a behavioral intervention that involves the structured practice of complex cognitive activities such as memory training, problem-solving, and visual search skill training, all of which can be delivered with mobile phones [6]. It is one of the few behavioral interventions shown to be beneficial for maintaining or improving cognitive function in older adults with intact cognition or mild cognitive impairment [7-9]. Scalability is a major advantage, as the widespread use of smartphones and tablets offers an unprecedented opportunity to deliver cognitive training interventions remotely to a large population at a relatively low cost. This can be particularly beneficial to the immigrant populations, as they often rely heavily on smart devices to build social networks and access local services [10,11].

Despite the potential advantages of mobile-based cognitive training, most prior interventions have been tested exclusively in non-Hispanic White older adults. Currently, no tailored cognitive training program exists that can accommodate the unique immigration history, cultural values, and linguistic characteristics of older Chinese Americans. Additionally, adherence to cognitive training interventions is challenging [12,13]. A recent large-scale cognitive training trial reported that nearly 40% of the participants did not complete any of the training, which may be due to the complexity and intensity of the cognitive training activities [12]. Nevertheless, prior research has suggested that social support and a positive attitude toward the study are potential facilitators that can help older adults adhere to cognitive training interventions [12,14]. Therefore, engaging end users early in all phases of intervention development is critical not only for facilitating treatment adherence but also for achieving sustainability, broad dissemination, and implementation.

To engage end users early, experience-based co-design offers a novel approach that combines participatory research and end users’ experiences to improve care delivery [15]. Specifically, this approach focuses on users’ experience and an equitable partnership between researchers and users with the aim of identifying challenges and co-developing practical solutions [15,16]. In the context of developing a cognitive training intervention for older Chinese Americans, the experience-based co-design approach allowed us to incorporate older Chinese Americans’ prior knowledge, lived experiences, and social norms around dementia and cognitive aging into the intervention development. In doing so, the co-developed cognitive training intervention is expected to be not only culturally relevant but also hold great potential to generate a positive impact on maintaining cognitive function in older Chinese Americans.

To address this gap, we co-designed a mobile-based cognitive training intervention with older Chinese Americans and their adult children to ensure cultural and linguistic relevance. We aim to pilot-test this cognitive training intervention to evaluate its feasibility and acceptability. We also aim to gather preliminary effect sizes for outcomes including global cognition, mental health, physical functioning, and quality of life to inform the design of a future large-scale study. We hypothesize that by co-designing with end users, this cognitive training intervention will be culturally relevant, highly feasible, and acceptable among older Chinese Americans.

## Methods

### Study Design

This is a National Institutes of Health (NIH) Stage 1 [17,18] pilot randomized controlled trial (RCT) with a 12-week duration. Throughout the intervention development process, we leveraged our strong partnerships with a local Chinese community organization and industry partner and applied experience-based co-design principles. All participants (N=30) will be randomly assigned to a cognitive training intervention group or a waitlist control group on a 2:1 ratio through a computerized program [19]. Feasibility, acceptability, global cognition, mental health, physical functioning, and quality of life will be measured at baseline, 8 weeks, and 12 weeks.

### Ethical Considerations

This study was approved by the Duke Health Institutional Review Board (Pro00109934) and registered at ClinicalTrials.gov (NCT05355870). Signed informed consent from participants will be obtained before recruiting them into the study. All identifying information of the participants will be anonymized and kept confidential through the assignment of a unique study ID.

### Participants

We will recruit 30 older Chinese Americans without cognitive impairment in the RCT. To be eligible for the study, participants must meet the following inclusion criteria: (1) self-identify to be of Chinese descent, (2) are age 60 years or older, (3) are fluent in written and spoken Mandarin Chinese/English, (4) have the visual capacity to read a smartphone/tablet screen and the auditory capacity to understand normal speech (can be with glasses or hearing aids), (5) have no self-reported diagnosis of ADRD or cognitive impairment and are capable of making an informed consent, and (6) do not plan to move outside of the Research Triangle Park area of North Carolina in the next 6 months. Individuals are excluded from participation if they (1) are bedridden, (2) are receiving chemotherapy for malignancy, or (3) have other life-limiting illnesses.

### Sampling and Recruitment Strategy

During the initial phase of the study, we worked closely with community stakeholders to develop and refine our sampling and recruitment strategy. Specifically, we held multiple meetings with community leaders from large local Chinese community organizations including the Chinese American Friendship Association of North Carolina (CAFA-NC) and Asian Focus to discuss the sampling and recruitment process. By working with the Duke Recruitment Innovation Center and these key Chinese community stakeholders, we co-developed the recruitment flyers and other materials to ensure their cultural relevance. Then, by leveraging our strong partnership with CAFA-NC, we implemented the following key strategies to recruit participants:

1. Connecting with key community stakeholders to understand the catchment areas and characteristics of older Chinese Americans residing in North Carolina
2. Liaising with local leaders in the Chinese community, such as the president of CAFA-NC, organizers of local older Chinese American clubs, and founders of local Chinese schools to increase awareness about our study
3. Posting study flyers on local Chinese media sites, Chinese community organization newsletters, and social media groups such as WeChat groups and Facebook communities
4. Collaborating with a local senior center to conduct community outreach programs to promote awareness of brain health and our study

Preliminary data suggested that these recruitment strategies worked well among older Chinese Americans, as more than 100 eligible participants signed up for the study within 1 day after the first posting of study flyers. In addition, through co-design activities, we identified a few additional strategies that we may implement if needed. For example, some older adults suggested reaching out to older Chinese Americans at local Chinese social events (eg, Dragon Boat Festival). Moreover, a few local Chinese community organizations expressed willingness to make the initial contact and introduce this study to eligible participants.

### Experience-Based Co-Design Activities

During the first phase of the intervention development, we conducted 4 focus group discussions with older Chinese Americans (n=21) to elicit their dementia knowledge and prior experience with cognitive training, mobile games, and so on. Recognizing that support from the adult children of older Chinese Americans may facilitate engaging older Chinese Americans in cognitive training, we conducted another 2 separate focus groups with adult children (n=9) for additional input. We carried out a rapid analysis [20] of the focus groups and identified potential design issues with the intervention. Detailed results from the focus group discussions were published elsewhere [21].

In the meantime, we also built connections and worked with the Posit Science team to co-develop a linguistic and cultural adaptation protocol. As a digital health company specializing in providing brain training software and services, Posit Science offers BrainHQ, a mobile-based cognitive training app that comprises over 30 cognitive training exercises in English. These trainings target various cognitive domains, including memory, attention, and visual and auditory processing speed, and have been widely tested and implemented in prior research [22-24]. Through an iterative process guided by the Framework for Reporting Adaptations and Modifications-Expanded [25], we worked with scientists and technicians at Posit Science to make adaptations that translated its cognitive training components to ensure its linguistic and cultural relevance to older Chinese Americans. We also conducted internal user testing and used the results to further refine the cognitive training app in Mandarin Chinese.

Based on the findings from focus group discussions and the adapted Chinese version of BrainHQ cognitive training exercises, we conducted a co-design workshop with a series of activities that included a journey map and prototype development with 10 older Chinese Americans and 4 adult children. The goal of the co-design workshop was to engage end users and learn from their experiences to improve the delivery of the BrainHQ cognitive training program to older Chinese Americans and make it culturally relevant. These co-design activities were developed based on the Experience-Based Co-Design method and our prior experience [16]. Specifically, we first provided each participant with access to all the cognitive training exercises in the adapted Chinese version of BrainHQ. A few weeks before the workshop, the participants were asked to try out each cognitive training exercise and encouraged to participate in training as much (or little) as they would like to. Then, the participants were invited to the half-day in-person co-design workshop to provide feedback and develop potential approaches to address the identified design issues and strengthen the intervention components.

During the co-design workshop, we first introduced the goal of the co-design workshop and explained the process. In addition, we set ground rules to encourage rich, supportive discussions and for participants to feel valued and heard. In the first part of the workshop, we co-created a user journey map [26] with participants to systematically capture the entire process, experience, and emotions of end users when completing the cognitive training in the adapted Chinese version of BrainHQ. To ensure that participants felt comfortable and empowered to map the journey of the cognitive training together, we first introduced an example that mapped out the process of catching a flight. We then asked the participants to consider what could be improved for each step of the end users’ experience of the cognitive training. Discussion points were written by the facilitator on Post-it notes and stuck to a whiteboard for visualization during the workshop. Results from prior focus group discussions were also presented to participants and incorporated into the discussion points. During the workshop break, the research team organized and synthesized key points from the journey-mapping discussion into workable issues. In the second part of the workshop, the team asked participants to collaborate with each other and the research team to come up with solutions and co-design intervention components to address these issues (prototype development).

During the discussion, the research team mainly functioned as the facilitator to guide and redirect the discussion as needed. Participants were asked to suggest potential solutions and provide additional feedback and new ideas. At the end of the workshop, we debriefed with participants about the accomplishments of the co-design activities and explained the next steps of the research. Our research team then discussed the findings and finalized the intervention delivery plan, incorporating the participants’ input. Table 1 presents the identified workable issues and strategies co-designed with participants and refined by the research team after the workshop.

**Table 1:** Identified design issues and co-designed strategies.

Design issues	Strategies
How can we best convey perceived susceptibility and benefits to Chinese older adults?	Provide a brochure with updated scientific information on ADRD^a^ from credible sources.
How can we best engage participants’ families and leverage a sense of family responsibility?	Inform family members about the study. Make family members aware of the training schedule. Encourage family members to remind participants as they see fit.
How can we best build self-efficacy when using the app?	Provide information on the purpose of each training (eg, targeting which cognitive skill). Help participants interpret the results from each training exercise.
What would be the ideal frequency, intensity, and duration of the training?	Participants prefer a shorter duration with more intense frequency. Do not set an upper time limit for training.
How can we make the training more enjoyable and ensure adherence?	Allow participants to choose training exercises by themselves. Provide a wide array of training exercises. Create an online training community to promote a sense of belonging.

^a^ADRD: Alzheimer disease and related dementias.

### Co-Developed Intervention

Based on the feedback from the co-design workshop, participants in both the intervention group and the control group will be provided with a brochure that includes information on ADRD, signs and symptoms related to ADRD, the definition and the potential benefits of cognitively stimulating activities for maintaining cognitive function, and examples of cognitively stimulating activities. The brochure was developed based on materials from organizations such as the Alzheimer’s Association, the National Institute on Aging, and the World Health Organization (WHO), with additional feedback from our co-design workshop. Participants in the intervention group will complete a series of cognitive training exercises on a smartphone/tablet.

By co-designing with older Chinese Americans and their adult children, we propose a 12-week training schedule that involves daily training sessions that last between 15 and 20 minutes. The frequency and duration of the proposed training are also consistent with most prior interventions that implemented training lasting 20 to 60 minutes per session, about 3 to 7 times a week for a total of 6 to 12 weeks [8]. We recommend that each session include 2 training exercises that target different cognitive domains. Participants can go beyond the recommended training plan and complete more exercises as desired. Table 2 presents the exercises chosen by participants from the co-design workshop that are available in the adapted Chinese version of BrainHQ. As participants progress, the difficulty level will be automatically adapted based on their performance.

**Table 2:** Selected cognitive training exercises by cognitive domain through the co-design workshop.

Cognitive domain and training exercises		Description
**Attention**		
	Target tracker	Track moving objects on your screen.
	Double decision	Recognize and memorize the type of car and the location of the Route 66 road sign.
	Divided attention	Sort objects based on the instruction as quickly and accurately as you can.
	Mixed signals	Hear and see 2 different pieces of information and decide if the 2 pieces of information match.
	Freeze frame	Differentiate target images from other images.
**Processing speed**		
	Eye for detail	Identify where identical images appear on your screen.
	Sound sweeps	Memorize and recognize the sound tone based on what you heard.
	Visual sweeps	Determine whether each one is swept inward or outward.
	Fine-tuning	Differentiate between 2 words with similar sounds.
**Memory**		
	Scene crasher	Memorize the location of a specific key among several keys showing on the screen.
	Memory grid	Find and match cards of the same words among cards with words that have similar sounds.
	Hear, hear	Hear and remember the target tone and then identify the target tone among a set of tones.
**People skills**		
	Face facts	Remember the names, faces, and facts of a group of people.
	Recognition	Recognize the target face among a set of faces.
	Face-to-face	Understand and match facial expressions.
**Intelligence**		
	Mind bender	React to rapidly changing rules.
	Card shark	Memorize the sequence and information of a series of cards.
	Juggle factor	Place a series of numbers and shapes in the correct order.
**Visual special ability**		
	Right turn	Mentally rotate images to decide if they are identical or mirrored.
	Mental map	Remember and recall the relative location of objects in a grid.
	Optic flow	Match the shape and color of the sign while driving on the road.

### Waitlist Control

We will use a passive control in this pilot trial, as previous intervention studies and meta-analyses have suggested no differences between active and passive control groups [27-29]. Participants in the control arm will complete all assessments at time points corresponding to the intervention group but will otherwise continue to receive their usual care. At the end of the study, participants in the control group will receive access to the adapted Chinese version of BrainHQ cognitive training exercises.

### Co-Developed Study Procedures

After enrollment, participants will complete a baseline assessment administered by a bilingual and bicultural research assistant (RA) in person or via Zoom. The assessment will consist of sociodemographic characteristics (eg, age, gender, marital status, living arrangement, and education), acculturational factors (eg, years in the United States, English proficiency), lifestyle factors, medical history, and dementia knowledge (via the Alzheimer’s Disease Knowledge Scale) [30,31]. Participants in the intervention group will be instructed by the RA to create a user account for the adapted Chinese version of BrainHQ and access the training exercises. Additionally, the participants will choose a preferred time in the day to complete training and receive a training reminder. To ensure that the participants fully understand the intervention, the RA will use didactic instruction and roleplay to demonstrate the use of the Chinese version of BrainHQ. Participants will also be provided with a brief introduction and video demonstration of each cognitive training exercise through the BrainHQ app. Additionally, the RA will explain how to interpret the results from each training exercise. Based on the feedback from the co-design workshop, the RA will help form an online social group among participants in the intervention group, with their permission, aiming to create an environment for participants to support each other during the training and foster a sense of belonging. To ensure consistency with the intervention protocol, the following actions will be taken: (1) we will develop a manual for the intervention setup, which the RA will follow; (2) the training schedule and exercises will be preprogrammed into the app; and (3) ratings of treatment adherence and competence will be recorded and evaluated. Over the study period, the RA will interact with the participants only if they experience technical difficulties or have an extended period of training absence (missing 3 sessions in a row). If participants miss an entire week’s training, the RA will call the participant to determine the reason and potentially help resolve some of the issues. Participants who do not have a smartphone or tablet will be equipped with internet access. A separate masked RA will perform outcome assessments.

### Outcome Assessments

Our primary outcomes are feasibility and acceptability. Acceptability will be assessed using the Client Satisfaction Questionnaire (CSQ) [32,33]. Feasibility will be determined based on study accrual, protocol adherence, and retention. Specifically, accrual will be indicated by meeting the recruitment goal of 30 eligible participants in 6 months. We will use descriptive statistics to assess the number of older Chinese Americans reached and the rate of eligibility and response rate. Adherence will be assessed based on the proportion of participants in the intervention group who successfully completed all intervention sessions and the proportion completing the 8- and 12-week assessments in both arms. Additional performance measures will be assessed by exploring the usage data to capture the average time per day that participants spend on cognitive training exercises and the percentage of days that participants use the adapted Chinese version of BrainHQ. Trajectory modeling (eg, mixed models) with an emphasis on data visualization will be used to describe the BrainHQ usage over the intervention period. Retention will be assessed by the percentage of participants remaining in the study at the end of 12 weeks.

In addition to feasibility and acceptability, we will assess the following outcomes at baseline, 8 weeks, and 12 weeks. The assessment time points were chosen because prior research has suggested a significant difference in improvement between those who trained <8 weeks and 8 weeks [8]. Global cognition will be assessed using the Mini-Mental State Examination [34,35], Digit Span Test [36], verbal fluency [37], and the Trail Making Test [38]. As in the prior research, we will calculate the *z* score of each test and use the average *z* score to measure global cognition [9,39]. Mental health will be assessed with the (1) Patient Health Questionnaire-9 [40,41] for depressive symptoms, (2) UCLA 3-item Loneliness Scale [41,42] for loneliness, and the (3) Hospital Anxiety and Depression Scale-Anxiety Subscale [43,44] for anxiety. Physical functioning will be assessed with the Activities of Daily Living and Instrumental Activities of Daily Living Scale [45,46]. Quality of life will be assessed using the WHO Quality of Life Instrument-Abbreviated Version [47-49]. All study measures have a validated Chinese version and have been widely used in community-based studies among Chinese older adults.

### Data Analysis

For our primary outcome, we consider 70% completion as the benchmark for adherence. If fewer than 40% of participants complete all the cognitive training and assessments, the proposed intervention will be considered as not feasible. Acceptability will be assessed using the CSQ, and 80% of the participants in the intervention arm reporting satisfaction will suggest good acceptability. Trajectories of BrainHQ usage over the intervention period will be described, which may be helpful in recognizing changes in training intensity and identifying strategies to improve adherence in future studies. We will calculate between-group effect sizes to evaluate the effects on improving global cognition, mental health, physical functioning, and quality of life. Between-group effect sizes will provide an index of relative changes for intervention vs. control participants.

### Sample Size Considerations

Our target enrollment is 30 individuals over a 6-month period. A power calculation will not be appropriate as the primary purpose of this pilot RCT is not to evaluate statistical significance [50,51]. Rather, we are evaluating the feasibility and acceptability of our cognitive training intervention and obtaining preliminary effect size estimates to power a future study. We anticipate that the sample size of 30 participants will provide sufficient input to inform intervention refinement and improvement for a larger RCT under the limitations of time and funding resources.

## Results

This pilot trial received institutional review board approval in November 2024. We enrolled the first participant in December 2024. As of February 2025, we have enrolled 15 participants and are aiming to complete enrollment by May 2025. We expect to complete all data collection by September 2025. We will analyze the data and report study findings by February 2026.

## Discussion

### Expected Findings

We hypothesize that this co-designed cognitive training intervention is not only highly feasible and acceptable but also holds great promise to promote long-term adherence among older Chinese Americans. More specifically, we aim to achieve our previously stated goals by having at least 70% of participants complete the training and at least 80% express satisfaction with the intervention. Because this is a feasibility study with a relatively small sample size, we do not expect to see significant differences in cognition, mental health, and physical function measures between the intervention and the control groups at the end of the study.

From the co-design workshop, our team gained experience working with the targeted population and obtained valuable feedback on the best way to engage with the participants in addition to seeking their input on the study design. This workshop also served to raise awareness of dementia prevention and as a channel to outreach to the target population. This innovative co-design approach for engagement with older Chinese Americans was feasible based on our study and can potentially benefit other similar studies in the future.

### Comparison With Prior Work

Compared to prior cognitive training interventions [8,52], our study is among the few that apply the experience-based co-design approach. As prior studies have shown low adherence to cognitive training intervention [12,13], engaging older adults throughout the intervention development process can help optimize adherence to the training regime.

Prior research has also suggested that targeting older adults who are able to use mobile devices might be a limitation. However, our preliminary data from this study and prior work have suggested that smart device ownership is high (n=23, 91.2%) in this population [53]. In addition, we have prepared smart device rentals for any participants who do not have a smartphone/tablet equipped with internet access and need one. We will monitor for participants’ differences in enrollment/refusal to assess whether digital literacy is a potential barrier for older Chinese Americans to participate in cognitive training.

### Strengths and Future Directions

Our study aimed to address the issue of inadequate accessibility of high-quality cognitive training adapted for older Chinese Americans. As a critical first step (NIH Model Stage 1) in behavioral intervention development, the findings from this study will lead to our future work of testing the efficacy of this cognitive training intervention on cognition as well as physical and mental health in older Chinese Americans (Stage 2).

We also expect our work to have positive impacts on health promotion in the broader communities. With support from the CAFA-NC and Posit Science, this project provides an innovative partnership opportunity among academic, local community, and industry stakeholders. With support from our industry partner and community stakeholders, the research infrastructure created by this project is expected to produce more innovative mobile health (mHealth) interventions that can be scaled up rapidly in the future. While the research will be conducted on older Chinese Americans, this cognitive training intervention has the potential to be translated to other vulnerable populations that are traditionally excluded in dementia research (eg, other Asian Americans and Chinese older adults living in Asia)**.**

### Study Limitations

This pilot RCT may have the following limitations. First, the sample size for this RCT is relatively small, and we may not be adequately powered to detect statistically significant differences in outcomes between the intervention group and the control group. However, the primary objective of this pilot RCT is to establish the feasibility and acceptability of the cognitive training intervention through co-designing with end users. In addition, we recognize that some older Chinese Americans may speak Cantonese rather than Mandarin Chinese. Due to limited resources, we were not able to develop a complete set of cognitive training exercises in Cantonese. However, a few Cantonese-speaking participants mentioned at the co-design workshop that they had no issue understanding Mandarin Chinese. Finally, the pilot trial is taking place in the Research Triangle Park area of North Carolina. In recent years, North Carolina—particularly the Wake, Durham, Orange, and Mecklenburg counties—has become the second most popular destination for Asian immigrants, resulting in a large, growing, and socioeconomically diverse Asian immigrant population [54]. The lessons and experiences learned from this geographic area may not be applicable to older Chinese Americans living in other geographic regions such as traditional ethnic enclaves (eg, Chinatowns in the Northeast). However, our team has established a strong partnership with local Chinese community organizations in New York City and the Dallas-Fort Worth area as well, which will allow us to conduct a large-scale multisite intervention study to test the real-world efficacy (Stage 3) in the future.

### Dissemination Plan

We will report on the intervention development, methods, and results from this project by submitting manuscripts to major scientific and biomedical journals aimed at reaching the widest possible audiences. Additionally, we will actively seek opportunities to disseminate our findings through presentations to a variety of audiences. This will include presentations at major Alzheimer disease and aging conferences. In addition, the media relations offices at Duke will help accelerate the dissemination of our findings. Most importantly, we will work closely with our community partner CAFA-NC to report the progress and results of this study to participants and the local Chinese community via community-wide newsletters and social media posts.

## Appendix

Multimedia Appendix 1 Peer review report from the HPC - Health Promotion in Communities Study Section, Healthcare Delivery and Methodologies Integrated Review Group (National Institutes of Health, USA).

## Abbreviations

ADRD: Alzheimer disease and related dementias
CAFA-NC: Chinese American Friendship Association of North Carolina
CSQ: Client Satisfaction Questionnaire
mHealth: mobile health
NIH: National Institutes of Health
RA: research assistant
RCT: randomized controlled trial
WHO: World Health Organization

## Data Availability

All data are presented in the main manuscript.
