# Supplementary material for: Co-Designed Mobile-Based Cognitive Training for Older Chinese Americans: Protocol for a Pilot Randomized Controlled Trial Assessing Feasibility and Acceptability
Source: JMIR Res Protoc. 2025 Jul 21;14:e69303. doi: 10.2196/69303 (PMC12322612; doi:10.2196/69303)
Supplement: Multimedia Appendix 1 [file resprot_v14i1e69303_app1.pdf]

1R21AG075228-01 Xu, Hanzhang

RESUME AND SUMMARY OF DISCUSSION: This application proposes to use an experience-based co-design approach to collaborate with older Chinese Americans, industry partners, and community stakeholders and develop a mobile health (mHealth) intervention for cognitive training that is culturally and linguistically tailored to older Chinese Americans. The rigor of prior research is strong and the panel agreed that the project's high significance lies in its ability to advance understanding on effective culturally tailoring mHealth intervention to maintain cognitive status among older ethnic minorities facing increased rates of Alzheimer's Disease and related dementias (ADRD). The research environment is excellent and the investigative team is strong with relevant expertise and experience working with the proposed study population. The panel found several strengths with the approach, including grounding in the Health Belief Model to guide development and adaptation of the intervention and engagement of community and business partners in the development of the app. The involvement of both older Chinese end users as well as their children in the development of the app is also a strength because culturally, elderly Chinese may depend on children for daily activities. This particular consideration bodes well for adherence and retention. The panel noted a few weaknesses with the approach, including lack of information on the technological literacy and use of ZOOM or mobile apps in the study population. There is also lack of engagement with evidence indicating that cognitive training programs like the one proposed are not very effective in delaying cognitive decline. The selection of 30 participants for the pilot trial lacks justification. An initial difference of opinion among panel members was largely based on the differential weighing of strengths and weaknesses in determining the project's impact rather than on any substantive disagreement. Reviewers who weighed the application more favorable emphasized the exploratory of the mechanism and felt the high significance and stellar investigative team and environment would lead to success. Following discussion, the difference in opinion was slightly reduced. Overall, findings are likely to have a moderately high impact on the development and implementation of effective mHealth interventions that promote cognitive aging tailored for older Chinese Americans and other ethnic minority older adults.

DESCRIPTION (provided by applicant): Older Chinese Americans are one of the fastest growing populations in the United States, who face many social and economic barriers. Over 70% of older Chinese Americans are first generation immigrants, nearly 20% are living below the poverty line (9% in general US older adults), and close to half of them have limited English proficiency. These socioeconomic disadvantages shared by older Chinese Americans will not only contribute to an increased risk of developing Alzheimer's disease and related dementias (ADRD), but also inequitable access to effective strategies to promote cognitive health. Cognitive training is one of the few interventions shown to be beneficial to the maintenance or enhancement of cognitive function in older adults with normal cognition or mild cognitive impairment. The widespread use of mobile phones offers an unprecedented opportunity to deliver cognitive training interventions remotely to a large population at relatively low cost. This is particularly relevant to the immigrant populations as they often reside in ethnic enclaves and rely heavily on smartphones and apps to build social networks and to access local services. Despite the promise of mobile app-based cognitive training, most prior interventions were tested exclusively in non-Hispanic Whites. Currently, no tailored cognitive training program exists that can accommodate the unique immigration history, cultural values, and linguistic properties of older Chinese Americans. To address this gap, we aim to develop a mHealth intervention for cognitive training that is culturally and linguistically relevant to older Chinese Americans. We will leverage our strong partnership with a local Chinese community organization, industry partner, and interdisciplinary expertise in cognitive aging, ethnogeriatrics, implementation science, and experience-based codesign. More specifically, the proposed aims are: Aim 1: To adapt empirically supported cognitive training components into a culturally and linguistically relevant cognitive training intervention protocol. Aim 2: To conduct a pilot randomized controlled trial of the proposed cognitive training intervention to evaluate the

feasibility, acceptability, and obtain preliminary effect sizes on outcomes including global cognition (primary), mental health, physical functioning, and quality of life. This study will provide the scientific groundwork for a multi-site large scale randomized control trial to test the real-world efficacy of this cognitive training intervention. In addition, this project will further our knowledge of methods to support engagement of ethnic minority communities in clinical research studies. Taken together, these efforts are expected to ultimately facilitate the implementation of effective, sustainable, and scalable mHealth interventions to promoting cognitive aging specifically suited for ethnic minority older adults.

PUBLIC HEALTH RELEVANCE: Older Chinese Americans often have limited access to dementia care and prevention services and are not well- represented in dementia research. We propose to use an experience-based co-design approach to collaborate with older Chinese Americans, industry partner, and community stakeholders and develop a mHealth intervention for cognitive training that is culturally and linguistically tailored to older Chinese Americans. This study is a critical first step (NIH Stage Model Stages 0 & I) in behavioral intervention development and will lead to future work of testing the efficacy of this cognitive training intervention in older Chinese Americans and in other ethnic minority older adults.

CRITIQUE 1

Significance: 3
Investigator(s): 2
Innovation: 3
Approach: 4
Environment: 1

Overall Impact: The proposed application aims to adapt and pilot test a culturally relevant mHealth intervention for cognitive training to older Chinese Americans. The study is significant because it addresses an unmet need regarding maintaining cognitive status in older Chinese adults by adapting and pilot testing a culturally relevant mobile app based cognitive training program --- with input from older Chinese Americans and their adult children. However, the rigor of prior research is not fully addressed with a lack of data on a proportion of Chinese adults older than 60 years old (without upper age limit) using mobile App. This poses a question about the feasibility of the proposed intervention. The investigative team is well qualified to conduct the study with prior experience and collaboration in researching health problems among Chinese older adults. Creating a culturally relevant mobile app based cognitive training program --- with input from older Chinese Americans and their adult children --- is novel. The approach has several strengths including involving adult children in the study and conducting co-design workshops. Score-driving weaknesses include: (1) lack of data on the proportion of Chinese older adults able to use mobile App or zoom; (2) no psychometric data for Chinese versions of outcome measures; (3) no justifications provided for selecting 30 participants for a pilot trial and intervention control ratio of 2:1; and (4) sex as a biological variable is not considered. The study has a strong environment. Overall, the potential impact of this application is moderate.

1. Significance:

Strengths

- The proposed study addresses a growing public health concern of the burden of ADRD in ethnic minority older adults.

- The proposed study responds to an unmet need regarding maintaining cognitive status in older Chinese adults by adapting and pilot testing a culturally relevant mobile app based cognitive training program --- with input from older Chinese Americans and their adult children.

Weaknesses

- Lack of data on a proportion of Chinese adults older than 60 years old (without upper age limit) using mobile App poses a question about the feasibility of the proposed intervention.

2. Investigator(s):

Strengths

- The PI, early-stage nurse scientist has experience in studying cognitive aging in Chinese older adults and mixed-methods research
- The PI is supported by senior Co-Investigators who have experience in conducting research in Chinese older adults, gerontology, academic-community collaboration, co-design methodology, intervention development and implementation, and biostatistics.
- Senior research scientist at Posit Science will collaborate to provide technical support for using Brain HQ in Chinese.
- Some team members have a prior history of collaboration.

Weaknesses

- None noted by reviewer.

3. Innovation:

Strengths

- Creating a culturally relevant mobile app based cognitive training program --- with input from older Chinese Americans and their adult children --- is novel.

Weaknesses

- Other aspects of the study are not innovative.

4. Approach:

Strengths

- The study is grounded in the Health Belief Model.
- Including adult children in the study is a strength -- this is culturally relevant considering that elderly Chinese may depend on adult children on many daily activities.
- Conducting co-design workshops will be helpful to adapt and refine the intervention.

Weaknesses

Moderate

- Lack of data on a proportion of Chinese adults older than 60 years old (without upper age limit) using mobile App poses a question about the feasibility of the proposed intervention.
- Having zoom meetings with participants older than 60 may not be feasible if they do not have access to or do not know how to use zoom.

- No psychometric data for Chinese versions of outcome measures are provided.
- No justification is provided for selecting 30 participants for a pilot trial. Although this is a pilot trial, some justification for a sample size based on similar prior studies should be provided. It is questionable whether 30 may be sufficient even for a pilot trial.
- There is a lack of justification why intervention: control is 2:1 ratio.
- Sex as a biological variable is not considered.

Minor

- It is unclear if those who participated in focus groups will also participate in co-design workshop, or they will be excluded in the co-design workshop.
- It is unclear why a brochure is provided to participants when the intervention is provided through App.
- Training session of 3-7 times/week (especially 6-7 times/week) seems too much for elderly population.

5. Environment:

Strengths

- Resources provided at Duke University and New York University are adequate to complete the study.
- Letters of support include specific information about how support will be provided to the study.

Weaknesses

- None noted by reviewer.

Study Timeline:

Strengths

- The proposed timeline is appropriate.

Weaknesses

- None noted by reviewer.

Protections for Human Subjects:

Acceptable Risks and/or Adequate Protections

Data and Safety Monitoring Plan (Applicable for Clinical Trials Only):

Acceptable

Inclusion Plans:

- Sex/Gender: Distribution justified scientifically
- Race/Ethnicity: Distribution justified scientifically
- Inclusion/Exclusion Based on Age: Distribution justified scientifically

Vertebrate Animals:
Not Applicable (No Vertebrate Animals)

Biohazards:
Not Applicable (No Biohazards)

Resource Sharing Plans:
Acceptable

Budget and Period of Support:
Recommend as Requested

Recommended budget modifications or possible overlap identified:

- No budget allocation presented for Posit Science in budget justification, but letter of support included it.

CRITIQUE 2

Significance: 3
Investigator(s): 1
Innovation: 3
Approach: 2
Environment: 2

Overall Impact: This well-organized proposal seeks to develop a project to develop a cognitive training program for elder Chinese adults. The literature review establishes that Chinese American elders are at high risk for cognitive decline and lack access to cognitive training programs. The research plan is extremely well thought out and its many strengths include strong involvement from end-users and community members, an excellent partnership with local community organizations as well as a business partner, the use of mixed methods data collection and analysis. Weaknesses include the fact that cognitive training has not been completely established as an effective in delaying cognitive decline. The proposal does not review the negative findings in the literature and make a case for why and how their proposal should move forward in view of these findings. Also of concern is the fact that, according to the literature review, cognitive training programs have extremely low uptake and adherence. While it is highly appropriate for this proposal to engage users and adult children of potential users in modifying the intervention plan to increase acceptability and adherence in future users, the researchers do not provide any actual examples of the types of factors that might reduce acceptability nor the ways in which stakeholder input could be useful in increasing uptake. Still this is a well thought out proposal with potential high impact.

1. Significance:

Strengths

- Situates the proposed project in the context of the literature on previous cognitive training programs, highlighting the weaknesses of these programs

- Old Chinese Americans are fast growing, high risk group re: dimension and cognitive decline, but have limited access to dementia prevention
- Project aligns with WHO and NAM policies calling for more detention prevention interventions in low income communities of color
- Addresses current problems with adherence
- Appropriate pilot activities in preparation for a larger test of the intervention

Weaknesses

- Would have been helpful to understand more about the types of barriers to adherence that have been perceived in the past and examples of the types of information that might be generated via focus groups to address these barriers
- A broader concern is that cognitive training has not been established as an intervention that slows cognitive decline. There is a lack of a critical review of this literature and a placing of the current study as preparing for a better test in view of conflicting results.

2. Investigator(s):

Strengths

- PI is incredibly productive researcher, with numerous grants and publications, strong research and data analysis skills, and a commitment to the community. Other investigators are highly appropriate for this grant.
- Both industry and community partners are a strength

Weaknesses

- None noted by reviewer.

3. Innovation:

Strengths

- Development of a cognitive training app for Chinese elders is innovative, since this has not been done before
- Degree of close involvement of end users in ‘experienced based co-design’, including both adult children of older Chinese Americans and elders themselves, is unusual if not precisely innovative, and very noteworthy

Weaknesses

- Adaptation of an intervention for a new cultural groups is highly worth doing, but is not necessarily innovative

4. Approach:

Strengths

- The approach section is very strong. Selection of measures is closely tied to hypotheses.
- The research stages, moving from focus groups, to workshop, to small pilot, is highly appropriate
- Mixed methods data collection

Weaknesses

- It is not completely clear how the end user involvement will result in adaptations that could address the problems with adherence and retention that have faced other cognitive training programs. More details would have been beneficial.

5. Environment:

Strengths

- Excellent environment. The local population of Chinese elders is not very large, but the community relationships with the investigators suggest that there should be no problem getting the sample
- The Duke school of nursing is an excellent environment

Weaknesses

- None noted by reviewer.

Study Timeline:

Strengths

- Appropriate

Weaknesses

- None noted by reviewer.

Protections for Human Subjects:

Acceptable Risks and/or Adequate Protections

- no issues

Data and Safety Monitoring Plan (Applicable for Clinical Trials Only):

Acceptable

o no issues

Inclusion Plans:

- Sex/Gender: Distribution justified scientifically
- Race/Ethnicity: Distribution justified scientifically
- Inclusion/Exclusion Based on Age: Distribution justified scientifically
- no issues

Vertebrate Animals:

Not Applicable (No Vertebrate Animals)

Biohazards:

Not Applicable (No Biohazards)

Resource Sharing Plans:

Acceptable

- no issues

Budget and Period of Support:

Recommend as Requested

CRITIQUE 3

Significance: 2
Investigator(s): 3
Innovation: 2
Approach: 3
Environment: 1

Overall Impact: This application seeks to adapt a mHealth intervention for cognitive training for Chinese Americans using co-design and end-user strategies, then determine feasibility and acceptability via a pilot random controlled trial. The application leverages a strong local Chinese American community organization and an industry partner as collaborators. The proposed study has several strengths, including addressing the increase in Alzheimer's Disease and related dementias (ADRD), a growing problem in all populations; the issue of determinants of ADRD for ethnic minorities, including Chinese Americans; and the compelling need for culturally and linguistically tailored interventions to strengthen cognitive reserve in ethnic minorities. Further, the application includes a strong investigator team, led by a junior investigator with a growing research record, but balanced by senior co-investigators. The application includes a theory-driven process to adapt an established evidenced based intervention and a developmental process that includes cultural and linguistic approaches to garner input from older Chinese as well as adult children. The research environment at Duke University is especially strong for this application with established centers and institutes related to aging and health and the collaborating institution, New York University where Co-I Dr. Wu works, is equally strong. Developing a mHealth intervention for cognitive training may not be novel itself but the adaptation for an Asian American population is innovative. The application discusses how the intervention could be scaled for other Asian populations here as well as globally. A weakness in this application is the lack of clarity regarding the extent that ADRD is prevalent in the target population. More compelling data would have strengthened the justification for the study.

1. Significance:

Strengths

- This proposed study addresses Alzheimer's Disease and related dementias (ADRD), a growing problem in all populations, including Chinese Americans, the largest Asian population in the U.S.
- mHealth interventions via smart phones/tablets are a possible strategy to improve cognitive reserve and prevent ADRD

- Few culturally tailored interventions for cognitive training are available for ethnic minorities, especially Chinese Americans

Weaknesses

- It is unclear the extent that ADRD is prevalent in Chinese Americans

2. Investigator(s):

Strengths

- Strong investigator team with complementary strengths led by a junior investigator with a growing research record of grants and publications
- The team is balanced with senior investigators who bring considerable experience

Weaknesses

- None noted by reviewer

3. Innovation:

Strengths

- Although mHealth interventions on cognitive training are not new, the adaptation of the intervention using engagement strategies to produce a culturally and linguistically appropriate intervention is innovative and could be adapted for other ethnic minority populations

Weaknesses

- None noted by reviewer.

4. Approach:

Strengths

- Theory-driven conceptual framework guides the study and is incorporated in the adaptation process
- The engagement focus using co-design strategies and end user testing are particularly strong components in the approach
- Investigators have thought through possible problems and pitfalls and have developed contingency plans
- Study instruments are well-defined

Weaknesses

- One potential problem not identified is how study outcomes might be impacted by tech literacy and ability, especially in those who are loaned smart phones/tablets

5. Environment:

Strengths

- The research environment at both institutions is strong
- Duke University provides additional enhancements with its established centers and institutes supporting research related to aging and health

- The community partnership with the Chinese American organization appears to be well-developed and ongoing

Weaknesses

- None noted by reviewer.

Study Timeline:

Strengths

- The timeline appears reasonable

Weaknesses

- None noted by reviewer.

Protections for Human Subjects:

Acceptable Risks and/or Adequate Protections

Data and Safety Monitoring Plan (Applicable for Clinical Trials Only):

Acceptable

Inclusion Plans:

- Sex/Gender: Distribution justified scientifically
- Race/Ethnicity: Distribution justified scientifically
- Inclusion/Exclusion Based on Age: Distribution justified scientifically

Vertebrate Animals:

Not Applicable (No Vertebrate Animals)

Biohazards:

Not Applicable (No Biohazards)

Resource Sharing Plans:

Acceptable

Budget and Period of Support:

Recommend as Requested

THE FOLLOWING SECTIONS WERE PREPARED BY THE SCIENTIFIC REVIEW OFFICER TO SUMMARIZE THE OUTCOME OF DISCUSSIONS OF THE REVIEW COMMITTEE, OR REVIEWERS' WRITTEN CRITIQUES, ON THE FOLLOWING ISSUES:

PROTECTION OF HUMAN SUBJECTS: ACCEPTABLE

INCLUSION OF WOMEN PLAN: ACCEPTABLE

INCLUSION OF MINORITIES PLAN: ACCEPTABLE

INCLUSION ACROSS THE LIFESPAN: ACCEPTABLE

COMMITTEE BUDGET RECOMMENDATIONS: The budget was recommended as requested.

Footnotes for 1 R21 AG075228-01; PI Name: Xu, Hanzhang

+ Derived from the range of percentile values calculated for the study section that reviewed this application.

NIH has modified its policy regarding the receipt of resubmissions (amended applications).See Guide Notice NOT-OD-18-197 at https://grants.nih.gov/grants/guide/notice-files/NOT-OD-18-197.html. The impact/priority score is calculated after discussion of an application by averaging the overall scores (1-9) given by all voting reviewers on the committee and multiplying by 10. The criterion scores are submitted prior to the meeting by the individual reviewers assigned to an application, and are not discussed specifically at the review meeting or calculated into the overall impact score. Some applications also receive a percentile ranking. For details on the review process, see http://grants.nih.gov/grants/peer_review_process.htm#scoring.
